# Impact of the Egyptian summer season on oxidative stress biomarkers and some physiological parameters in crossbred cows and Egyptian buffaloes

**DOI:** 10.14202/vetworld.2018.771-778

**Published:** 2018-06-08

**Authors:** Maha M. Hady, T. M. Melegy, Shaimaa R. Anwar

**Affiliations:** Department of Nutrition and Clinical Nutrition, Faculty of Veterinary Medicine, Cairo University, Giza-12211, Egypt

**Keywords:** buffaloes, dairy cows, Egyptian’s summer, heat stress, oxidative stress

## Abstract

**Aim:**

The current study aimed to compare the impact of heat stress (HS) on some physiological functions and blood oxidative stress biomarkers between dry dairy crossbred (Balady X Friesian) cows and buffaloes during Egyptian summer season (July-September).

**Materials and Methods:**

A total of 26 healthy animals were equally used in the in the current study. The criterion for cows and buffaloes selection and the management conditions were similar. A total mixed ration to meet the animal’s requirements was used, and dry matter intake (DMI) was calculated. Ambient temperature, relative humidity, temperature humidity index (THI), respiratory rate, and rectal temperature (RT) were daily recorded. Meanwhile, live body weight and body condition score were weekly recorded. Blood samples were collected bi-weekly, and plasma samples were harvested for malondialdehyde (MDA) content and enzymatic antioxidants such as glutathione peroxidase, superoxide dismutase, and catalase activities determinations throughout the experimental period (8 weeks - prepartum).

**Results:**

The results confirmed, the HS condition, as the THI values ranged from 79.74 to 90.4 throughout the experimental period. In both species, HS increased RT and decreased DMI (<10.5 kg/day and 9.5 kg/day in cows and buffaloes, respectively). Buffaloes seemed to be more affected by the hostile environmental condition of this study compared with their respective cows. Buffaloes had recorded up to 1 °C increase in their RTs in most of the point’s period compared to cows. There was a continuous increase in MDA values (194.7 and 208.4 nmol/gHb in buffaloes and cows, respectively, 2 weeks prepartum) as the animals come close to parturition with moderate decrements for the enzymatic antioxidant activities in both cows and buffaloes.

**Conclusion:**

It can be concluded that during Egyptian’s summer season, HS had adversely affected feed intake and consequently animal’s production performances.

## Introduction

In general, Egypt’s summer is characterized by high ambient temperature and relative humidity (RH) which results in heat stress (HS) that ubiquitously affects the productive performance of livestock species. Oxidative stress (OS) is an increase in the generation of reactive oxygen species (ROS) more than the ability of the body antioxidant physiological mechanisms to do safe neutralization.

The earth’s climate has been predicted to change continuously at exceptional rates in recent decades [1]. Summer temperature in the Mediterranean region, including Egypt is generally outside of the cow’s “comfort zone” resulting in HS. The term HS is defined as the sum of heat accumulated from the environment and the failure of the animal to dissipate heat, which is mostly associated with a malfunction of the animal’s productive and/or physiological metabolic process [2]. Acute and chronic HSs exhibit different responses on animal’s production and metabolism. Temperature humidity index (THI) is a suitable measure to estimate HS in dairy cows [3]. It was suggested that THI can be classified into mild (72-80), severe (80-85), and deadly stress zones (>85) [4]. Increasing air temperature and the temperature-humidity index are accompanied by rising rectal temperature (RT) above critical thresholds which are related to a decrease in dry matter intake (DMI), milk yield, and reduced milk yield efficiency in dairy cows [5]. The magnitude of the animal’s response to elevated ambient temperatures depends on livestock species and their physiological state. Some species are reported to be the best tolerant to HS, namely goats [6], while pregnant and lactating ruminants are more susceptible to HS than non-pregnant and non-lactating ones. Glandular surface of sweat gland per cm^2^ of skin surface was 1.07 and 3.08 in cattle and that the skin thickness of buffaloes was about twice that of cattle, in Egyptian buffaloes [7]. The sweat glands in buffaloes are underdeveloped; this indicates that buffaloes have more poor heat tolerance than cattle (fewer sweat glands and black colored skin) and thus assigning buffaloes to be of reduced capacity to withstand HS and so need greater alertness to compete for such hostile condition [8]. Furthermore, HS is one of the wide varieties of factors which cause OS *in vivo*. OS results from increased production of free radicals and ROS, and a decrease in antioxidant defense mechanisms [9]. The majority of the studies dealing with the effects of HS on OS biomarkers (cellular and molecular) response have been conducted in circumstances that sustained moderate to severe HS under strictly defined experimental conditions (environmentally controlled chambers) on selected high yielding and intensively managed dairy cattle. Scarce studies are available for the effect of moderate to severe HS on OS biomarkers (OSB) of dairy cows and buffaloes raised under Egyptian summer environmental conditions.

Therefore, the current study was planned to compare the impact of HS of the Egyptian summer conditions on some metabolic aspects and OSB of dairy cows and Egyptian buffaloes of moderate production.

## Materials and Methods

### Ethical approval

Care and management of experimental animals were done according to the guidelines of the animal care committee (CU -*II*-F-16-18) Cairo University.

### Animals, ration, and experimental design

A total of 26 healthy, mature animals (13 of crossbred Balady X Friesian cows and 13 Egyptian buffaloes) were equally distributed on the basis of multiparity (3-5 calving), expected calving date, previous lactation yield, body weight (BW), and body condition score (BCS 3.5-4.2) to an experimental trial during the dry period (8 weeks pre-parturient). Experimental animal’s average BW was 625±25 kg and the average fat-corrected milk (FCM) containing 4% fat was 19.40±2 kg for cows and 21.30±2 kg for buffaloes. FCM was calculated using the following equation: FCM = 0.4 * amount of milk + 15 * amount of fat [10]. Experimental animals were raised at a private farm in the Nobaria province during the period of the summer season (July-September). Animals were housed in shaded loose pens with adjacent outside yards supplied with evaporative mist and fan systems. The experimental animals were fed on commercially available total mixed ration. Clean water was supplied all the time. Daily DMI was recorded. The ingredients and calculated analyses of the ration are summarized in Table-1.

**Table-1:** Ingredients, nutrient’s composition, and calculated analysis of experimental ration.

Experimental ration (8 weeks prepartum)	% DM
Ingredient	DM
Corn silage	17.5
Beet sugar pulp	9.5
Yellow corn	18.4
SBM (44%)	10.2
Berseem hay	40.7
Salt	0.54
Sodium bicarbonate	1.00
Limestone	0.68
Calcium phosphate (monobasic)	0.50
Magnesium oxide	0.35
Premix*	0.13
Calcium bentonite	0.50
Total	100
Calculated analysis	
NE_l_ (Mcal/kg)	1.28
CP (% DM)	12.8
Forage NDF (% DM)	32.61
Ether-extract (% DM)	2.10
RUP (% CP)	33.45
Ca (% DM)	0.90
P (% DM)	0.37

* Contained 20.0% Cl, 13.0% Na, 10.0% Ca, 8.0% Mg, 8.0% S, 1.0% K, 0.62% Zn, 0.54% Mn, 0.20% Fe, 0.08% Cu, 0.07% I and 0.2% Co, 551 IU/g Vitamin A, 132 IU/g Vitamin D, 3 IU/g Vitamin E. NE_l_=Net energy for lactation, CP=Crude protein, NDF=Neutral detergent fiber, RUP=Rumen un-degradable protein, DM=Dry matter

### Measurements and sampling

#### Environmental temperature and humidity

Digital hygrometer-thermometer device was daily used for measuring RH and temperature (at 0600, 1200, and 1800 h) at a fixed time. THI was calculated according to Thatcher *et al*. [11].

#### Animal temperature and respiratory rate

RTs and respiratory rates were obtained twice daily (at 0600 and 1600 h) with a digital rectal thermometer and visual counting flank movements during a 15 S. interval and then multiplied by 4.

#### Live BW and BCS

BWs and BCS measurements were done weekly by the same technician for all animals immediately at morning and before feeding.

#### Blood sampling and analysis

Bi-weekly blood samples were collected from all animals (cows and buffaloes) during the 8 weeks prepartum-2 weeks prepartum. All blood samples were collected from the coccygeal blood vessel then placed into 10-mL heparin Vacutainers. After proper centrifugation, the plasma samples were harvested and decanted into 1.5 mL aliquots and stored at −20°C until further analyses. Measuring OSB activity was determined through measuring plasma malondialdehyde (MDA) content, plasma glutathione peroxidase (GPX), catalase (CAT), and superoxide dismutase (SOD) activities using diagnostic kits supplied by Biodiagnostic^®^ Company, Egypt. Plasma MDA content was determined according to the method of Draper and Hadley [12], and plasma GPX activity was estimated as described by Lin *et al*. [13]. CAT and SOD activities in erythrocyte were determined calorimetrically by the methods described by Goth [14].

### Statistical analysis

Statistical analysis of the data was assessed using Minitab 17.0^®^ statistical program [15]. Student’s t-test for independent groups was done. Significance was considered at p≤0.05 levels. Pearson correlation analysis between the effect of cows and buffaloes RT and DMI was also computed. In addition to that, predicted DMI in cows and buffaloes was obtained in relation to RT using line fit plot and regression equation. The significance of difference was based on the probability of a Type I error set at p≤0.05.

## Results and Discussion

The results of ambient temperature (T) (°C), RH and THI measurements throughout 8 weeks prepartum are presented in Table-2. The presented data revealed that the THI indices during the experimental period which lasted from July to September had reached the maximum 89.1 and 90.4 in the last 3 weeks prepartum. However, at all the times of the experimental period (Egyptian summer climate), the THI had reached more than 80 except for the 1^st^ week of the study (79.74). THI could be used as an indicator of thermal climate as indicated by Akyuz *et al*. [16]. According to Thatcher *et al.*, lactating cows are thought to experience no stress when THI is <72 and severe stress when THI exceeds 88 [11]. For each l0 L milk yield per day, metabolizable energy requirements of cows are roughly doubled, and nearly 35% of this energy is dissipated as heat [17]. Hence, high yielding cows suffer more than lower yielding ones, because the upper critical temperature shifts downward as milk production, feed intake, and heat production increases [18]. Nevertheless, buffaloes have been reported to be more susceptible to HS than cows, as the increase in temperature causes stress due to increased body heat loading and the low potentiality to dissipate heat from the body surface due to fewer and ill-developed sweat glands as well as black colored skin [19]. THI values more than 72 are considered stressful, and THI over 78 is recognized as very severe HS to buffaloes [20]. The degree to which an animal resists change in body temperature varies in different species because of differences in their heat-regulating mechanisms [21].

**Table-2:** Average atmospheric temperature (°C), RH (%), and THI during 8-week prepartum (July-September).

Weeks prepartum	T	RH	THI
8	31.6	50.2	79.74
7	36.2	50	85.9
6	37.4	51.2	87.1
5	37.8	49.8	87.9
4	38.4	49.7	88.8
3	39.1	52.4	89.1
2	38.7	50.2	89.1
1	39.7	51.2	90.4
Average	37.7	50.2	87.2

RH=Relative humidity, THI=Temperature humidity index

**Table-3:** Average weekly RT (°C) and respiratory rate (breaths/min) of experimental animals during the dry period (8 weeks prepartum).

Weeks prepartum	RT		Respiratory rate	
	
	Cow	Buffalo	Cow	Buffalo
8	39.1	40.5	70.5	71.8
7	38.7	39.8	69.5	68.7
6	38.5	38.7	63.4	62.8
5	39.2	40.3	67.4	67.2
4	39.4	40.8	67.8	68.1
3	38.7	40.2	65.2	66.4
2	39.8	40.7	67.9	68.4
1	40.1	41.1	71.8	72.6
t-value	0.88	1.07	2.41	3.73
Significance	NS	NS	NS	NS

NS=Non-significant at P>0.05. RT=Rectal temperature

**Figure-1:**
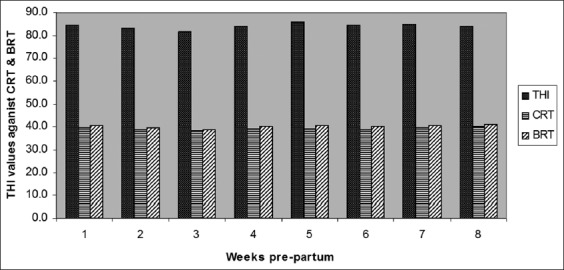
Impact of temperature humidity index on cows and buffaloes rectal temperature (°C).

**Figure-2:**
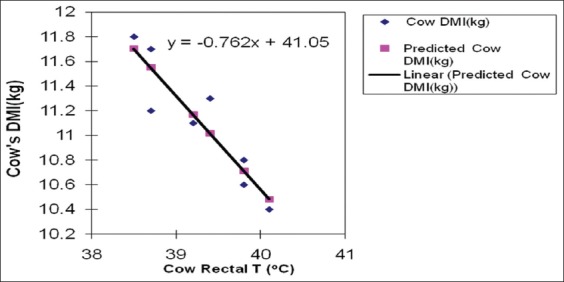
Regression analysis between cow rectal temperature (°C) and dry matter intake (kg).

**Figure-3:**
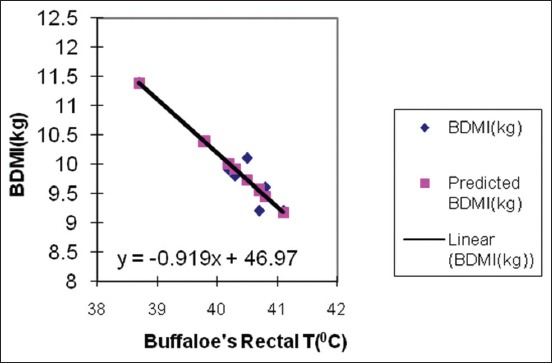
Regression analysis between buffaloes rectal temperature and dry matter intake.

RT is a reliable tool to determine animal’s temperature followed by ambient temperature. The results of the impact of Egyptian summer conditions on RTs and respiratory rates of cows and buffaloes during 8 weeks prepartum are presented in Table-3. The results demonstrated that there was no significant difference between species, although both cows and buffaloes were severely affected by HS as the recorded RT values were higher than normal values in the thermoneutral periods. However, buffaloes seemed to be more affected by the hostile environmental condition of this study compared with their respective cows. Buffaloes had recorded up to 1 °C increase in their RTs in most of the point’s period compared to cows. Similarly, Joshi and Tripathy recorded a 2.6°C rise in RT in buffalo’s calves when exposed to direct sun rays in the months of June and July [22] Air temperature (13-18°C), RH (55-65%), and wind velocity (5-8 km/h) are the optimum conditions for buffaloes and the THI ≥77 is very stressful [20]. On the contrary, Mullick reported that RT during the summer months under high and low humidity conditions was always less for buffaloes than cattle [23]. Singh *et al*. found that HS had a detrimental effect on lactation length, calving interval, milk constituents, and milk yield in Murrah buffaloes [24]. The relationship between THI as a measure of HS and animal RT (Figure-1) demonstrated that at the peak of severe HS, the THI was 89.1 and 90.4 attended during the last 3 weeks prepartum. Rectal body temperature is a sensitive indicator of physiological response to HS as it is nearly constant under normal conditions [25]. Regarding the respiratory rate (Table-3), there was no significant difference between species and both species attended the highest respiratory rates in the last week prepartum which is compatible with the results of THI and RT. HS significantly affected respiratory rate (breaths/min) when compared to normal values (76.8 and 77.8 vs. 40-50) for cow and buffalo, respectively, as presented by Das *et al*. [26]. According to Dalcin *et al.*, respiratory rate can be a relevant physiological indicator for HS [27]. A higher respiratory rate of 71.5/min during summer compared to 38.8/min in the winter was recorded in lactating cows by Silanikove [18]. Respiratory rate is an indicator of HS in the hot environment [27]. The normal respiratory rate is approximately 10-30 breaths/min [7]. There is a very high positive correlation between the respiratory rate and ambient temperature. The increase in breathing rate (panting) sharply increases the loss of CO_2_ through pulmonary ventilation with upsetting the critical balance of carbonic acid to bicarbonate excretion causing respiratory alkalosis [26]. In cattle under heat load, about 15% of the endogenous heat is lost directly from the body core through the respiratory tract [28]. HS that is characterized by elevated respiratory rates and RTs has been implicated in impaired metabolism and in poor reproductive performance in dairy cattle as well as in dairy buffaloes [26,29] independent of any effects on feed intake. The results of regression analysis between cows and buffaloes RTs and DMIs are presented in Figures-2 and 3, respectively. Predicted DMIs in cows and buffaloes were obtained in relation to RT using the following models: y=−0.7624x+41.057 and y=−0.9195x+46.972, respectively. Where: y = variable measured (DMI) and x= RT taking into account that the following models are applied when RT as well as THI from 38 to 41°C and 79-90, respectively. In addition, the regression coefficient for buffaloes and cows is −0.9195 and −0.9196, respectively. It was also noticed that with the increase in the RT there was a decrease in the DMI in both species. The increase of the RTs of the cows over 40°C was associated with a dramatic decrease in the DMI to <10.5 kg/day. However, buffaloes demonstrated higher RTs, especially in the last week prepartum which was associated with a DMI drop to <9.5 kg/day. The significant negative correlation between animal’s RT and its DMI was previously reported in Holsteins [25]. According to Rhoads *et al.*, feed intake started to decrease at air temperatures of 25-26°C in dairy cows and dramatically declined above 30°C in moderate climatic condition and at 40°C [30], it may decrease by as much as 40%. Moreover, the same correlation was demonstrated in buffaloes as up to 40% reduction in voluntary DMI during the summer months was recorded as compared to the amount consumed during the cooler months [31]. Feed intake reduction due to the increase in temperature might be attributed to several reasons, among them the direct effect of elevated temperature on the appetite center in the hypothalamus through increasing leptin and adiponectin hormones levels [32], resulting in reduction of the production of volatile fatty acids which are the main energy source in ruminants [33]. Feed intake reduction can also occur as a result of the inverse relationship between DMI and non esterified fatty acids concentrations during the peri-parturient period [34].

The results of the impact of HS on OSB of dry cows and buffaloes are presented in Table-4. It was observed that the values of MDA (nmol/gHb) as an indicator of OS ranged from 112.12 to 194.7 and from 125.87 to 208.4 in cows and buffaloes, respectively, during the 8 weeks prepartum period. There was an insignificant difference between species; however, buffaloes exhibited the highest values, especially in the last 2 weeks prepartum. Moreover, the MDA content in both species demonstrated marked increase toward the calving date (2 weeks prepartum) as the metabolic load increased. Mitochondrial membrane constituents are particularly susceptible to oxidative damage by ROS. Phospholipid constituents of the mitochondrial membranes are rich in unsaturated fatty acids which are susceptible to the attack by oxygen radical, due to the presence of double bonds, which undertake peroxidation through a chain of oxidative reactions [35]. MDA is an end product due to polyunsaturated fatty acid peroxidation. High plasmatic MDA levels occur due to HS [36].

**Table-4:** Different stress biomarkers of cows and buffaloes during Egyptian summer at different weeks prepartum.

Species	Weeks prepartum							

	8				6			
	
	MDA	GPX	SOD	CAT	MDA	GPX	SOD	CAT
Cow	112.23±10.4	38.14±1.1	6.25±0.52	26.71±2.4	123.4±8.47	37.65±2.1^a^	7.14±0.81	26.14±3.10^a^
Buffalo	125.87±8.6	32.62±1.8	7.48±0.64	25.63±1.4	132.33±9.7	30.41±1.4^b^	6.85±0.52	22.27±1.9

**Species**	**Weeks prepartum**							

	**4**				**2**			
	
	**MDA**	**GPX**	**SOD**	**CAT**	**MDA**	**GPX**	**SOD**	**CAT**
Cow	165.84±9.7	29.4±1.1^a^	5.11±0.51	21.3±2.6	194.7±17.2^a^	28.7±1.8^a^	3.49±0.42	18.41±1.7
Buffalo	185.61±10.2^a^	25.7±0.7^b^	4.48±0.32	18.6±1.9	208.4±16.4	22.9±1.3^b^	3.56±0.51	14.31±1.32

Designation of the values of MDA as nmol/gHb, GPX as mol/mg Hb and SOD and CAT as U/mg Hb, values with different superscripts at the same column significantly differ at P<0.05. GPX=Glutathione peroxidase, MDA=Malondialdehyde, SOD=Superoxide dismutase, CAT=Catalase

These results indicate that despite the metabolic stress that occurred in the pregnant dry animals which were aggravated by the HS; it could adjust to the environment by secreting large quantities of cortisol [24]. Higher concentration of this catabolic hormone normally results in lipolysis, and adipose mobilization in heat stressed cows to initiate and maintain milk production [37]. Similar to our results, several studies demonstrated that the MDA content increased around calving [38,39].

The results of the enzymatic antioxidant capacity of dry cows and buffaloes during summer season (Table-4) revealed that there was an insignificant difference between species except for the GPX starting from 6 weeks prepartum. One can observe that as the animals come near to calving time, the levels of the enzymatic antioxidants exhibited modest instable decrements. The average values recorded for GPX, SOD, and CAT in cows and buffaloes are within the values recorded by other studies [40,41], respectively. The modest decrease in the enzymatic antioxidant activity was in harmony with the confirmed HS condition observed in the current study. Megahed *et al*. examined the effects of HS in Egyptian buffaloes in summer and winter seasons and reported that SOD activities were significantly lower in the summer as compared to the winter season [41]. On the contrary, some authors demonstrated a significant increase in SOD and CAT in buffaloes [42] and cattle [38] during summer compared to the spring season. The enzymatic antioxidants, mostly metalloenzymes are the first line of a defense system that counteracts the oxidative damage of the intracellular constituents induced by ROS [26]. The discrepancy of the enzymatic antioxidant activity results might be attributed to multi-factors at these particular points among them: The physiological status of the animal (heifer, dry, or first trimester of milk production); type of HS (experimental chambers vs. field situation) as well as the methodology used. One possible explanation that both species were subjected to long-term HS at stressful metabolic and physiological status (dried pregnant animals) which was accompanied by marked reduction in DMI. This reduction in DMI (low nutrients and antioxidant intakes) plus persisting secretion of catabolic cortisol to satisfy energy needs had resulted in lipolysis, which eventually increased the production of free radicals and ROS and exhausted the antioxidant defenses.

## Conclusion

It is to be concluded that the Egyptian summer environmental condition is incriminated for HS and using THI is a good indicator. As high yielding animals, moderate yielders are also affected by HS. RT and DMI are highly correlated to HS. Dairy buffaloes seem to be more affected by HS than cows. As the field of OS in ruminant medicine is still in the early stages, the results of OS biomarkers furnish a base for such data, especially for buffaloes. An effective nutritional strategy by supplying surplus antioxidants to the ration of heat-stressed animal is a smart and effective promising path to alleviate metabolic, environmental heat loads.

## Authors’ Contributions

MM contributed in the design of the work, data collection, and analysis, interpretation, drafting the article, revision, and final approval of the article to be published. TM contributed to the design of the work, data analysis and final approval of the article to be published. SRA helped in interpretation of the data, drafting the article, revision and final approval of the article to be published. All authors read and approved the final manuscript.
